# Detecting abnormality in heart dynamics from multifractal analysis of ECG signals

**DOI:** 10.1038/s41598-017-15498-z

**Published:** 2017-11-09

**Authors:** Snehal M. Shekatkar, Yamini Kotriwar, K. P. Harikrishnan, G. Ambika

**Affiliations:** 1 Indian Institute of Science Education and Research, Pune, 411008 India; 2 Department of Physics, The Cochin College, Cochin, 682002 India

## Abstract

The characterization of heart dynamics with a view to distinguish abnormal from normal behavior is an interesting topic in clinical sciences. Here we present an analysis of the Electro-cardiogram (ECG) signals from several healthy and unhealthy subjects using the framework of dynamical systems approach to multifractal analysis. Our analysis differs from the conventional nonlinear analysis in that the information contained in the amplitude variations of the signal is being extracted and quantified. The results thus obtained reveal that the attractor underlying the dynamics of the heart has multifractal structure and the variations in the resultant multifractal spectra can clearly separate healthy subjects from unhealthy ones. We use supervised machine learning approach to build a model that predicts the group label of a new subject with very high accuracy on the basis of the multifractal parameters. By comparing the computed indices in the multifractal spectra with that of beat replicated data from the same ECG, we show how each ECG can be checked for variations within itself. The increased variability observed in the measures for the unhealthy cases can be a clinically meaningful index for detecting the abnormal dynamics of the heart.

## Introduction

The complexity of many physiological rhythms originate from the underlying complex nonlinear dynamical processes^1–7^. The various levels of this complexity and their variations, if properly discerned, will be useful in understanding the abnormalities that lead to many pathological cases^8–10^. A mathematical representation of the complexity using dynamical equations is most often not realizable due to the many interacting variables and uncertain parameters involved^11,12^. Hence the only possibility to study the dynamics in such situations to is to rely on information that can be deciphered from signals obtained from these systems^13–15^. Over the last few decades, several such physiological signals like EEG, ECG, fMRI etc have been subjected to different techniques available under the broad area of nonlinear time series analysis^13,16,17^.

The dynamics of the human heart is well established as a complex system based on multiple approaches^18–20^. This complexity is naturally reflected in the ECG signal that is an average response of the dynamics of the heart. But cardiac abnormalities or malfunctions can cause subtle changes or variations that reduce its complexity^18,21^. Therefore, it is important to quantify the changes in complexity due to abnormalities in terms of variations in its measures. However, such complexity related quantifiers have not yet reached the clinics for effective diagnostics and therapy. For this, we have to develop a unique way of characterizing its complexity that will help to distinguish healthy and pathological cases. Moreover it is important that the method should be effective with the short ECG data available in normal clinical practices. In the present work, we report a study in this direction where measures derived from the multi fractal spectrum of ECG signals can be used as a promising tool in the diagnosis of abnormalities of the heart.

In this context we note that most of the reported research in related analysis^16,18,19,22–25^ are on the peak to peak time intervals (also called as *R-R* intervals)^26,27^ and hence do not include the possible information content in the amplitude variations and shape of the waveforms. Also the multifractal analysis of ECG data reported are mostly using the method of Detrended Fluctuation Analysis (DFA)^28^ which quantifies the variability in the scaling of the fluctuations in data.

We start with the hypothesis that even though ECG is an average response of the heart, the subtle variations in the actual shape of the ECG waveforms and their amplitude variations can be related to the variations in the underlying dynamics. Hence we follow a dynamical systems approach and try to reconstruct the attractor of the dynamics from the ECG waveform. We compute quantifiers that can capture the non-uniform distribution of points on this attractor that directly relate to its complexity and multifractal nature. Earlier studies based on multifractal analysis, using DFA or otherwise, mostly use the range of scales or width of the multifractal spectrum or its area as a characterizing measure indicating difference in complexity. In our study, we rely on at least four indices for a unique and complete characterization.

Over the last few decades, a wide variety of methods have been developed^29–35^ using the fractal measures of the embedded attractor. These methods have found successful applications in diverse fields like astrophysics^36^, physiology^22,37^, atmospheric sciences^38,39^, geology^40^ and stock markets^41^. Among them, an important class of methods is related to the characterization of the complex dynamics using multifractal analysis^13,42^.

We present the embedded attractors using Singular Value Decomposition (SVD) technique and establish the nonlinear nature of their dynamics using surrogate analysis with Correlation dimension as a discriminating measure. We compute the indices that characterize the multifractal spectrum uniquely and show how their ranges or variations can distinctly distinguish healthy and unhealthy sets. Applying the analysis to a large number of ECG data sets, we group the data into healthy and unhealthy sets based on the values of the indices. Using supervised machine learning approach, we indicate how a new data can be assigned to either of these groups with high accuracy.

In addition, we repeat the analysis using a set of beat replicated data generated from each ECG and their multifractal indices are compared with those from the full ECG. The variability in these is found to increase in the presence of any abnormality compared to healthy cases. This variability itself can thus be another clinically meaningful index for diagnosis of abnormality in heart dynamics from ECG data. The analysis presented is also advantageous over *R-R* interval or HRV analysis because the actual amount of time (one to two minutes) for which the ECG needs to be recorded is smaller by orders of magnitude. This will increase its applicability for the normal daily clinical purposes.

## Results

### Phase space reconstruction from ECG data

**Figure 1:**
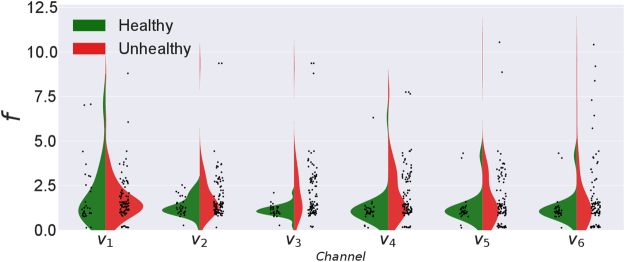
Violin-plot showing the distributions of frequencies with the maximum power for healthy and unhealthy groups for the six chest electrodes.

The data of 97 unhealthy and 32 healthy subjects obtained from PhysioBank database^43^ are pre-processed to make them suitable for the analysis (See Supplementary). Each dataset consists of ECG time series taken from six different chest electrodes or channels *v*_1_ to *v*_6_. As a preliminary analysis, we carry out the usual statistical and linear analysis and obtain the power spectra for all the data sets using the Fast Fourier Transform (FFT) algorithm. The frequency with the maximum power for each one of them and the distribution of these peak frequencies for all the subjects are summarized in Fig. 1 for data from six chest electrodes. It is clear that it is not possible to conclusively distinguish the two groups using the peak frequencies as they fall in almost similar ranges.

**Figure 2:**
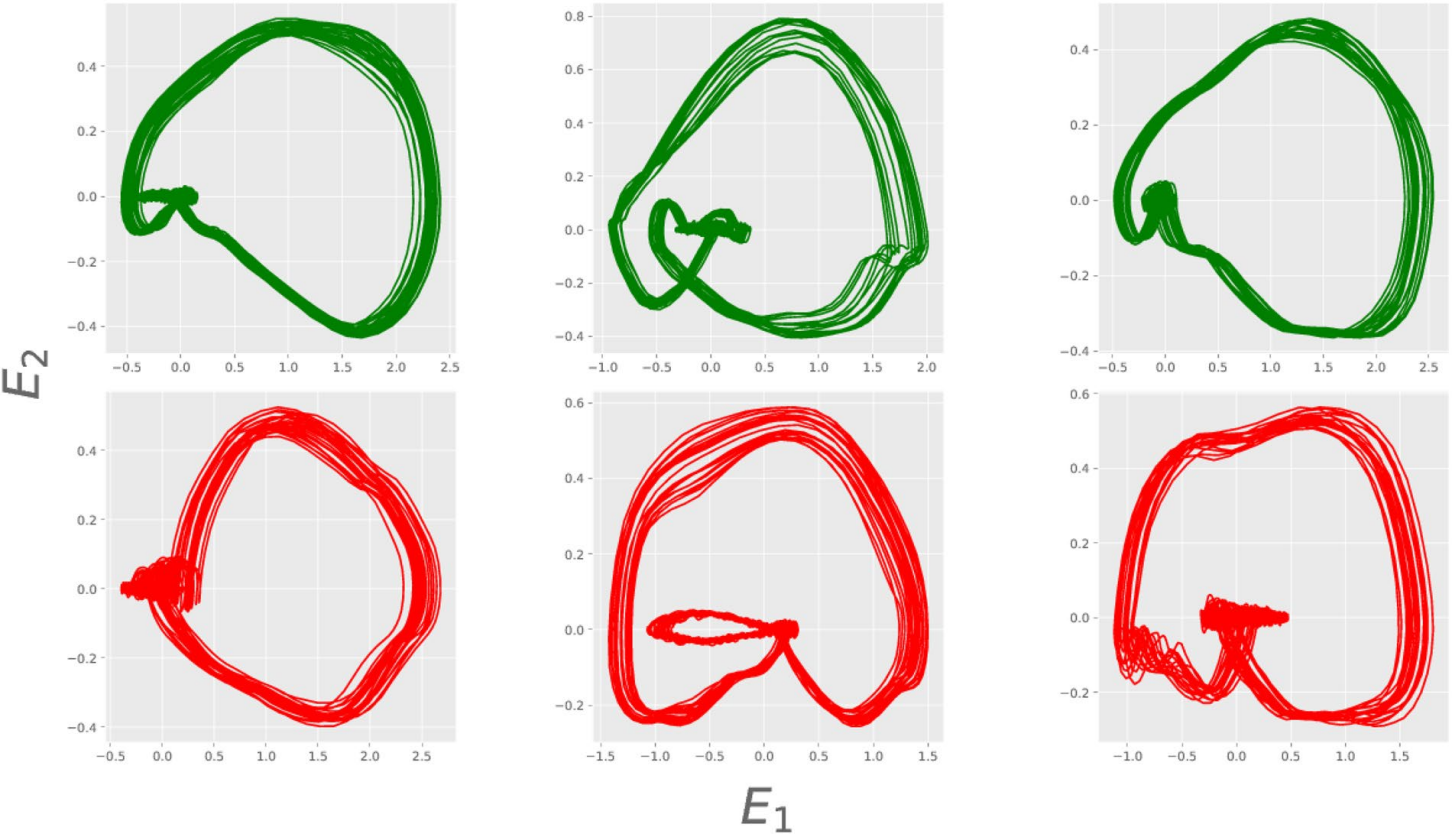
Two dimensional projections of embedded attractors for ECG signals for three healthy (top panel) and three unhealthy (bottom panel) cases. All the attractors are plotted using the SVD transformation.

Hence we resort to methods of nonlinear analysis and reconstruct phase space structure of the system’s dynamics from its discretely sampled time series *s*(*t*_*k*_). For a visual display of the resulting phase space structure or dynamical attractor, we use the technique of Singular Value Decomposition (SVD)^44,45^. (See Supplementary for details about embedding). In Fig. 2 we show a few representative embedded attractors from healthy and unhealthy groups. Each of the attractors is shown in the axes corresponding to statistically independent variables obtained from SVD.

Since the complex structure of the attractor inherently contains information about the complexity of its dynamics, it is important to characterize it quantitatively. Several quantifiers have been studied in this context over the years^46^, the most effective among them being the set of fractal dimensions. In the following sections, we indicate how correlation dimension is used along with surrogate analysis to establish the nonlinear nature of the underlying dynamics and how the geometrical complexity of its structure is quantified using multifractal measures.

### Correlation dimension and Surrogate analysis

**Figure 3:**
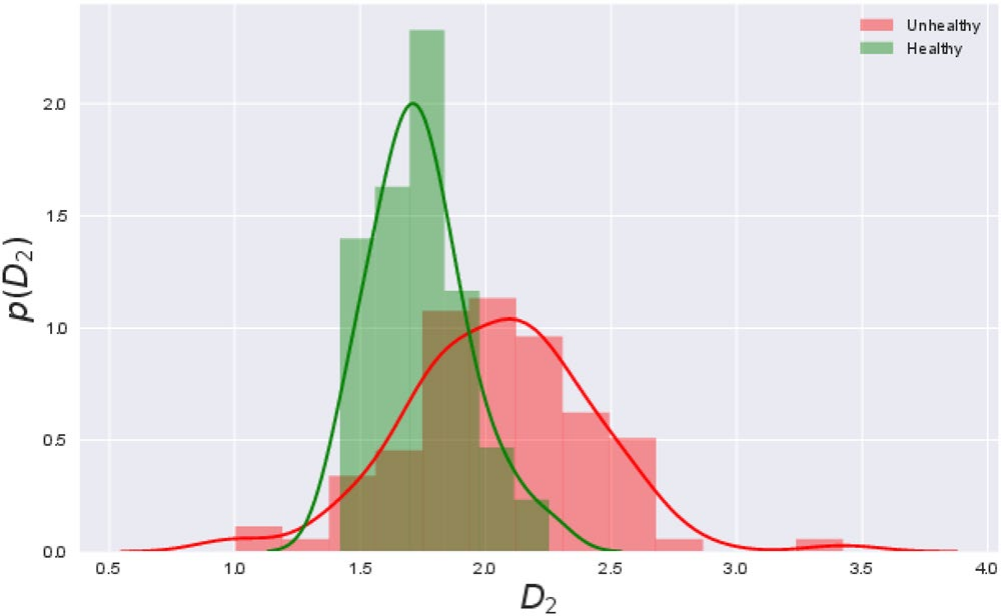
Distribution of correlation dimension (*D*_2_) values for healthy subjects (green) and unhealthy ones (red). As can be seen, all the values are less than 4 and so we choose *M* = 4 as the embedding dimension.

We recreate the phase space structure of the underlying dynamics in an embedding space of dimension *M* with delay vectors constructed by splitting the discretely sampled ECG data with delay time *τ*. The dimension *M* is chosen as the value at which any fractal measure, like correlation dimension *D*_2_, saturates^34^. The distributions of the saturated *D*_2_ values for healthy and unhealthy groups is shown in Fig. 3. Since all the *D*_2_ values are less than 4, for uniformity, we use *M* = 4 as the embedding dimension for all the signals.

**Figure 4:**
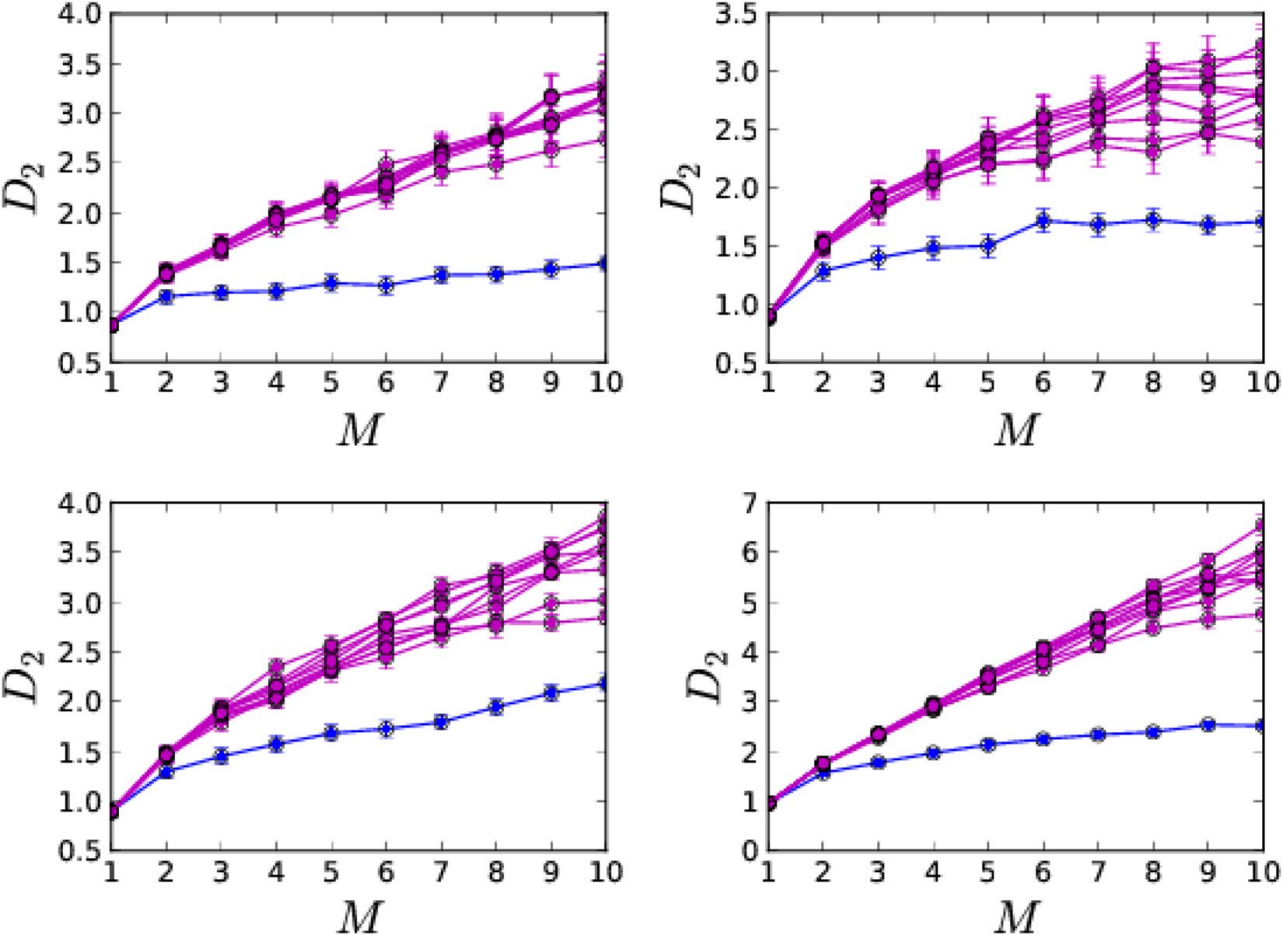
*D*_2_ values as a function of embedding dimension *M* for four randomly chosen datasets (blue) and their surrogates (magenta). It can be seen that the *D*_2_ values of surrogates sufficiently deviate from that of the original data.

Before undertaking any type of nonlinear analysis, it is important to first verify that the observed time series indeed results from an underlying nonlinear process. We check the nonlinear and deterministic nature of the underlying dynamics using a statistically rigorous method of surrogate analysis^31,32^. For this, we generate surrogate data using TISEAN package^47^. With correlation dimension as a measure, we plot in Fig. 4 the values for the original signal and the generated surrogates as a function of embedding dimension *M*. Since the values of *D*_2_ for surrogate data differ significantly from that of the original signal, we conclude that the signal comes from the underlying nonlinearity.

### Multifractal analysis

The reconstructed phase-space attractors for most of the complex systems like heart dynamics possess a multifractal structure which is characterized by a set of generalized dimensions *D*_*q*_ so that the non-uniformity in the distribution of points on the attractor becomes evident through different values of *q*. However the local scaling properties on the attractor are captured by a spectrum of singularities related to the probability measure on the course-grained attractor. Thus, if the attractor is covered by boxes of size *r*, the probability of points in the *i* ^th^ box scales as ${p}_{i}(r)\sim {r}^{{\alpha }_{i}}$. For a multifractal, the range of scales *α*_*i*_ present is a measure of its complexity. The number of boxes with the same *α* scales as ${N}_{\alpha }(r)\sim {r}^{f(\alpha )}$. Both (*D*_*q*_,*q*) and (*f*(*α*),*α*) provide analogous characterizations and are related by Legendre transformations as^35^:

1 $$\alpha =\frac{d}{dq}[(q-\mathrm{1)}{D}_{q}]$$

2 $$f(\alpha )=q\alpha -(q-\mathrm{1)}{D}_{q}$$

**Figure 5:**
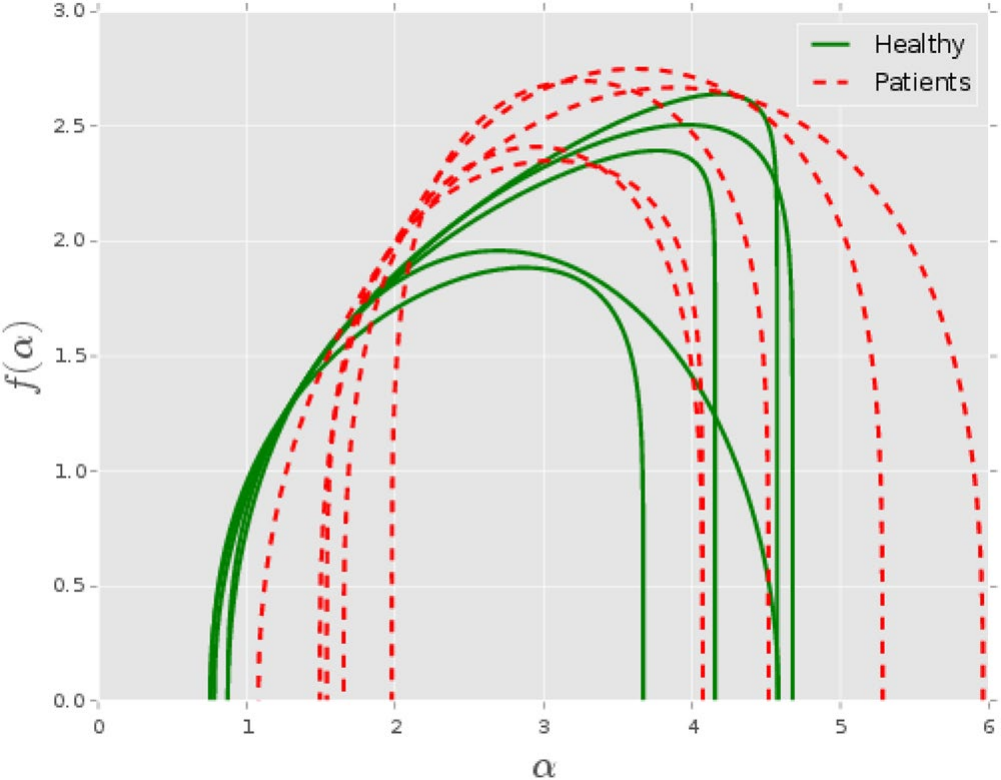
Multifractal spectrum curves for 5 randomly selected subjects from each group. The continuous green curves represent healthy subjects while the dashed red curves are for the non-healthy ones.

Thus, a convenient way of calculating the multifractal spectrum is to calculate the generalized dimensions *D*_*q*_ and then use equations (1) and (2) to find out *f*(*α*) and *α*. An algorithmic approach to perform this has been given by Harikrishnan *et al*.^35^ and here we follow the same method with suitable modifications for ECG signals. The multifractal spectra thus computed is shown in Fig. 5 for five randomly chosen data from both healthy and unhealthy groups. It is clear that the range of *α* values or the width of the spectrum and the shape of the curves vary between the two groups. These variations can be quantified to extract information on the variations in the complexity of the corresponding attractors. This is done with the following mathematical form for *f*(*α*) curve:

3 $$f(\alpha )=A{(\alpha -{\alpha }_{1})}^{{\gamma }_{1}}{({\alpha }_{2}-\alpha )}^{{\gamma }_{2}}$$

in which, as described in^35^, only four of the five parameters are independent. We choose the four parameters *α*_1_, *α*_2_, *γ*_1_ and *γ*_2_ to provide a unique characterization of *f*(*α*) spectrum of the multifractal.

**Figure 6:**
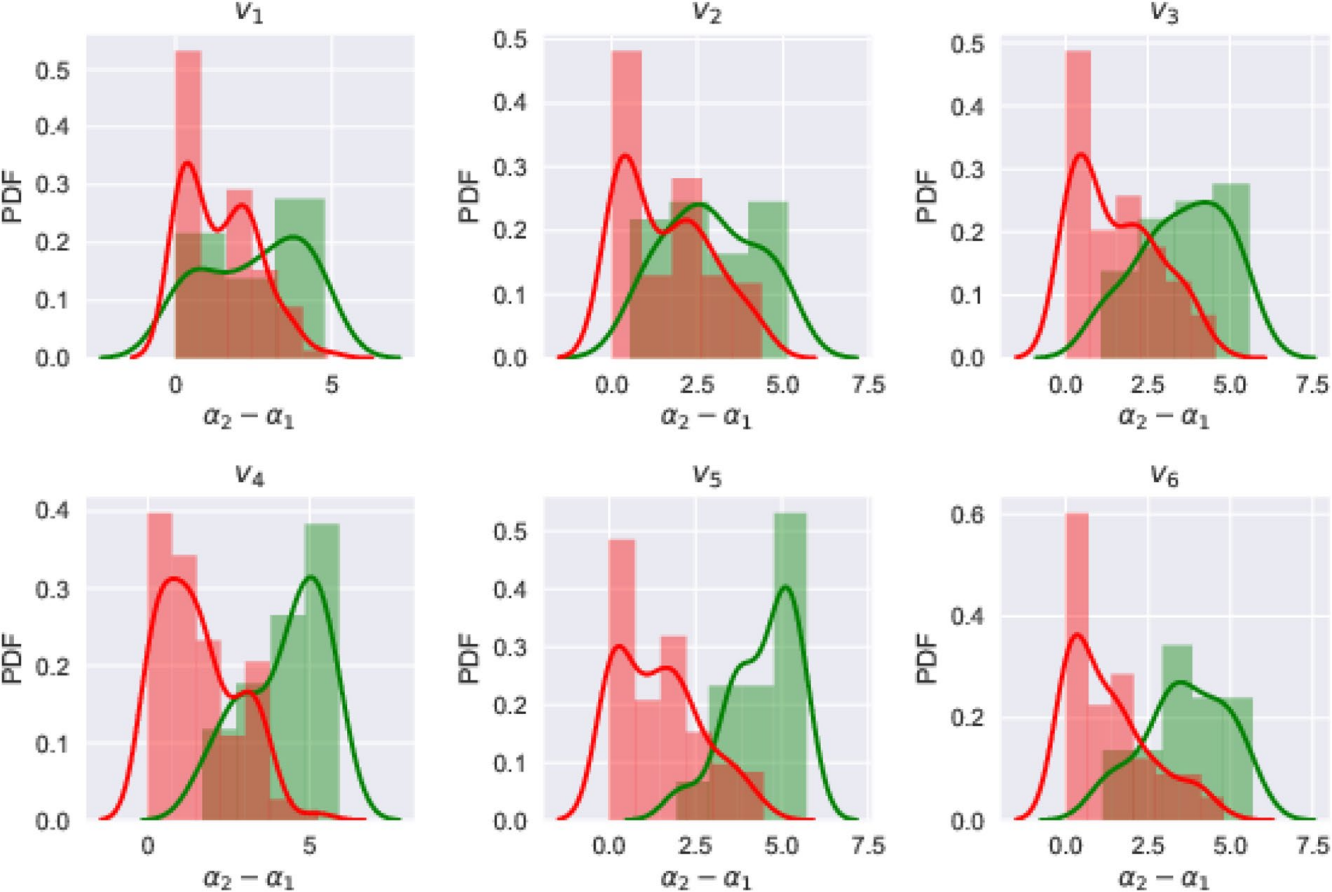
Distributions of the difference *α*_2_ − *α*_1_ for healthy(green) and unhealthy(red) groups for different channels. This difference is a measure of the complexity of dynamics underlying the ECG, as described in the text and thus, healthy hearts seem to be more complex than the unhealthy ones.

We note that the non-uniform distribution of the points on the embedded attractor indicates the complexity of the heart dynamics revealed through the ECG signal. Therefore, the range of scales required to describe the probability of the distribution of the points over the whole attractor becomes a measure of its complexity. The difference *α*_2_ − *α*_1_, that measures the width of the *f*(*α*) spectrum provides the range of scaling indices. In Fig. 6, we show the distributions of this difference for healthy and unhealthy groups. It can be concluded that the complexity tends to be more for healthy hearts across all the channels.

In principle, any of the above four parameters or any combinations of them should be a good indicator of the underlying complexity. For the data we have used, we find that the numerical error is more on the *α*_2_ side mostly because this corresponds to the sparse regions of the attractor. This can be overcome to some extent by using longer datasets. However, in the present study, we use only the ECG data readily available in normal clinical practices. Therefore, while deriving conclusions from the results, we concentrate on the *α*_1_ and *γ*_1_ values that are associated with the dense regions of the attractor as well as *α*_0_ the value of *α* corresponding to the maximum of the *f*(*α*) curve.

**Figure 7:**
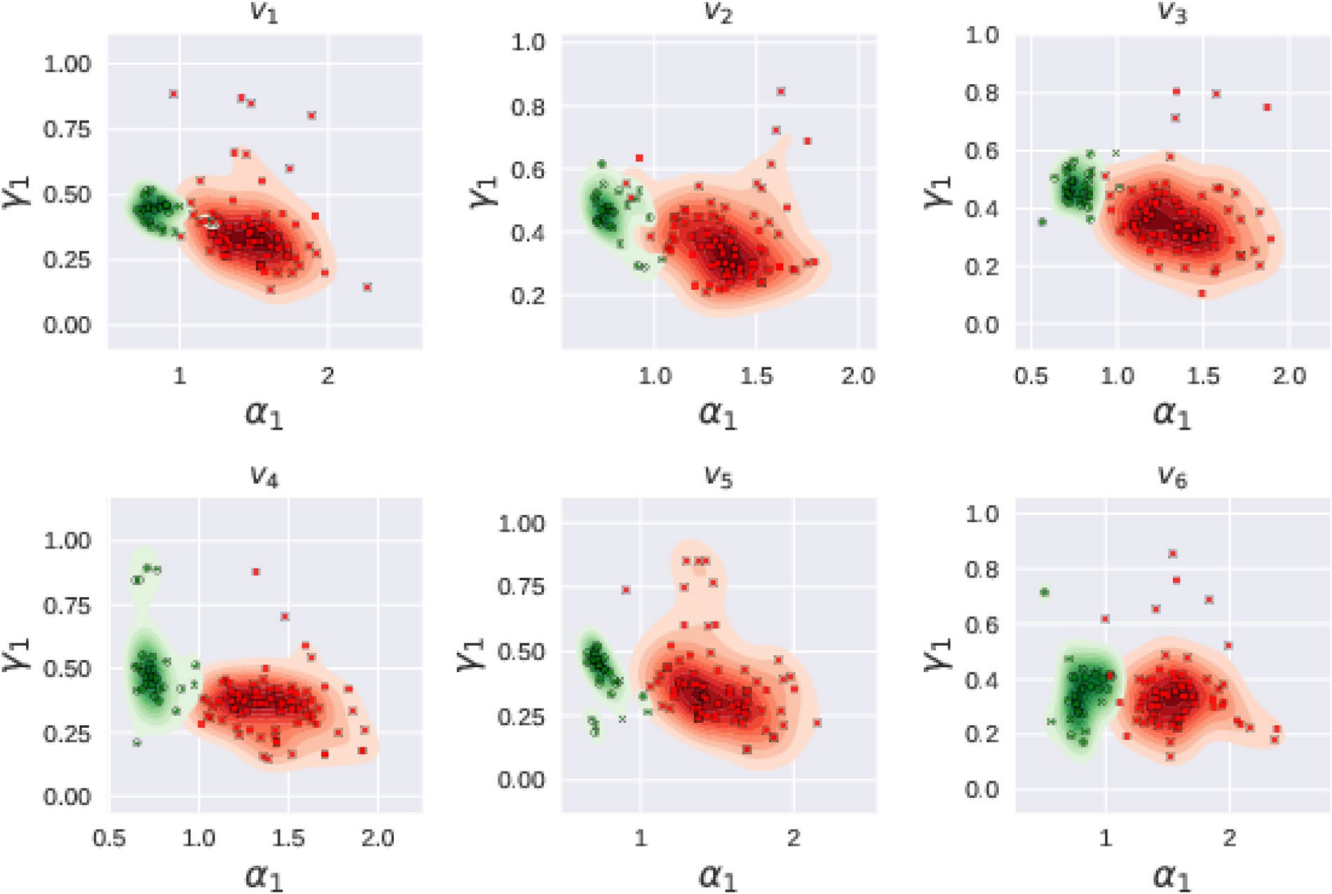
Scatter plots for electrodes *v*_1_ to *v*_6_ in *α*_1_-*γ*_1_ parameter planes. Green circles in the figure represent the healthy subjects and red squares represent patients. Overall, healthy cases are seen to be clustered whereas the patients are separated from healthy ones and are scattered.

In Fig. 7, we present the scatter plots of *α*_1_ and *γ*_1_ values for electrodes *v*_1_ to *v*_6_ obtained from the datasets from the two groups. The green circles in these plots represent the subjects identified as healthy in the PhysioNet database and the red squares represent the unhealthy ones. A few cases, for which the good fit could not be obtained have been discarded while plotting these parameter planes. For the purpose of visualization, we also show estimated kernel densities for the two groups as a background. As can be seen from these plots, multifractal analysis seems to have picked up almost every case correctly by separating the healthy and unhealthy cases into distinct clusters in *α*_1_-*γ*_1_ planes.

### Blind data testing and success rate

**Figure 8:**
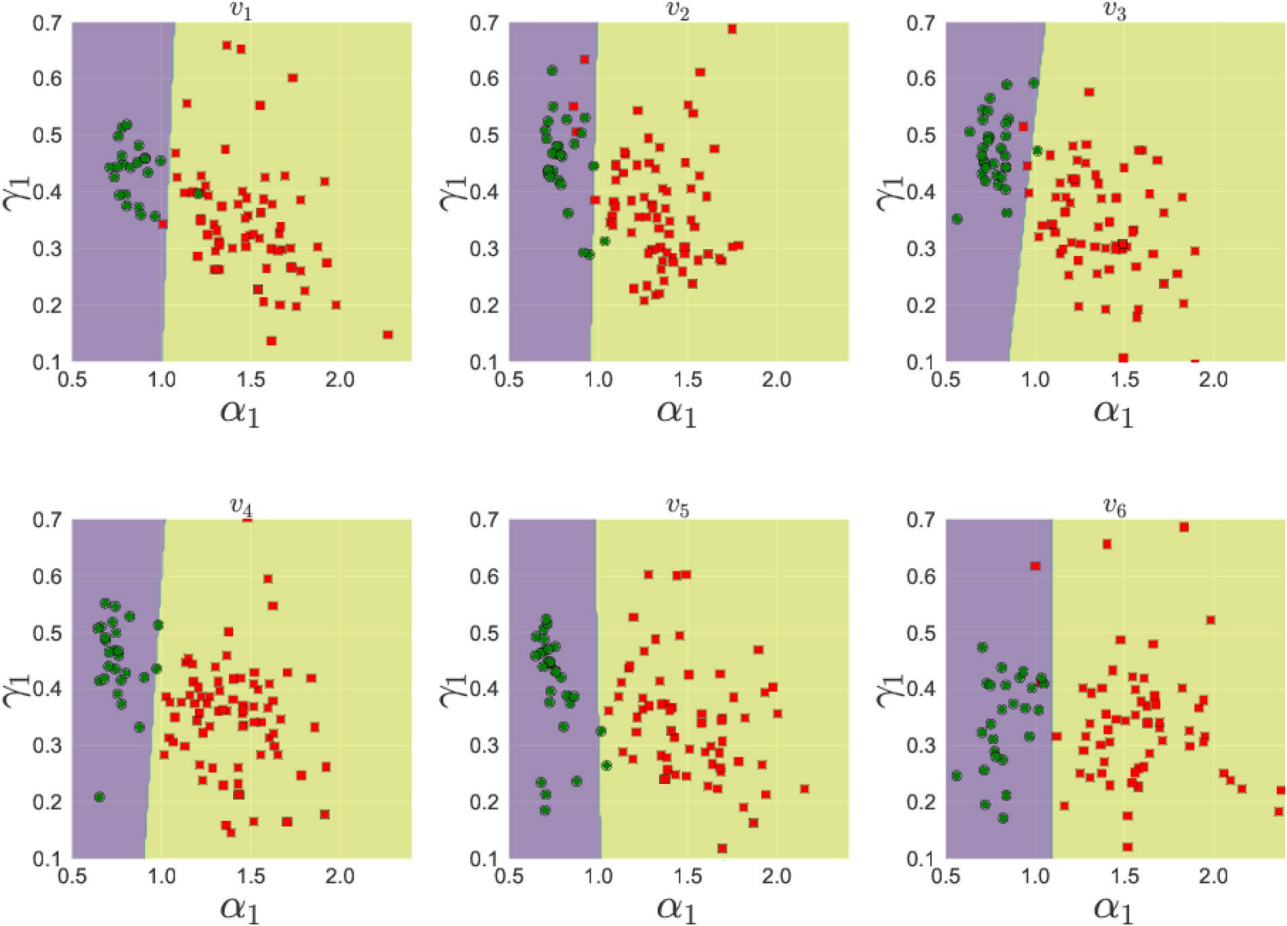
Regions of the parameter plane *α*_1_-*γ*_1_ that separate healthy and unhealthy regions obtained using SVC algorithm for electrodes *v*_1_ to *v*_6_. The actual data points are also shown for comparison with the regions.

**Figure 9:**
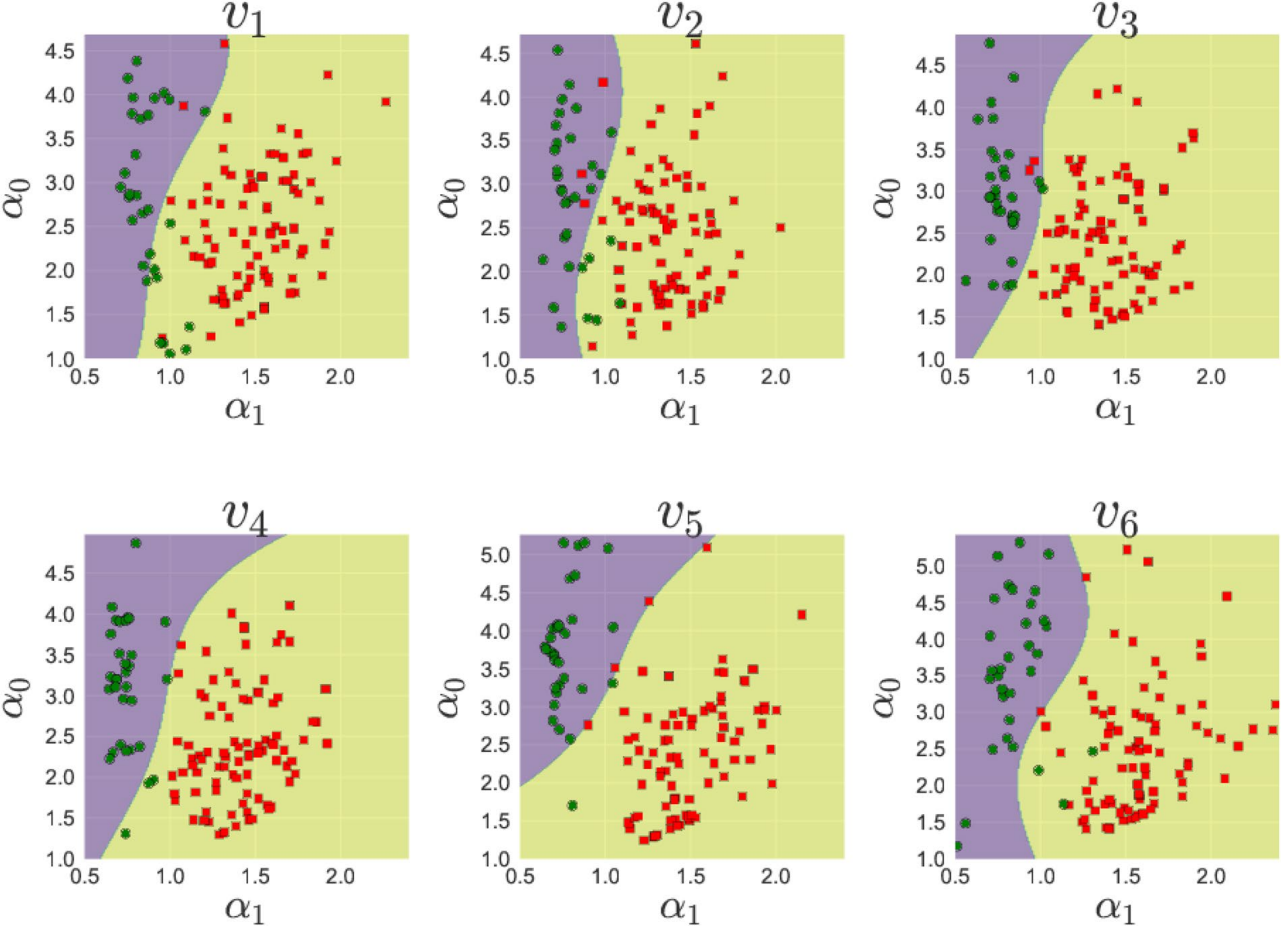
Regions corresponding to healthy and unhealthy cases in *α*_1_-*α*_0_ plane found using SVC algorithm.

The separation of the two groups into two different clusters becomes useful only if it helps us to predict the group label (healthy or unhealthy) for a new unseen data. This is a standard problem in the theory of machine learning and we use a particular algorithm called a “support vector clustering” or SVC using the Radial basis function (RBF) kernel^48^ to find out the regions in *α*_1_-*γ*_1_ planes corresponding to the two groups. The known group labels are used as a training data for the algorithm to identify healthy and unhealthy cases and then the algorithm is asked to divide the parameter plane into two regions. The regions so obtained for different channels are shown in Fig. 8. As another set of quantifiers, we now consider the parameter plane *α*_1_-*α*_0_, where *α*_0_ is the *α* value corresponding to the maximum value of *f*(*α*) curve and perform a similar analysis. The resulting regions are shown in Fig. 9.

**Figure 10:**
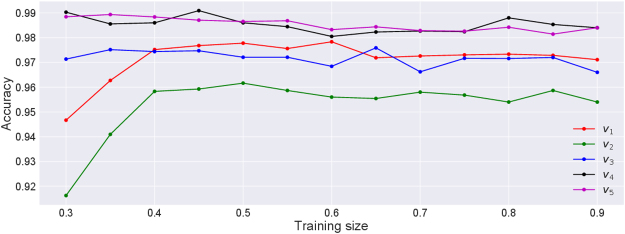
Accuracy of the SVC algorithm as a function of fraction of training data set. It can be seen that even for the low sizes of the training sample, the algorithm predicts the class labels with extremely high accuracy (>90%).

The regions shown in Figs 8 and 9 are obtained by training the SVC algorithm using the whole data. However, this does not tell us how well the algorithm would perform when given an unseen data. To check this, we split the whole data into two parts: training set and test set. We then train the algorithm on the training data and then ask it to predict the labels for the test data. To measure the success we calculate true positive rate (*t*_*p*_) and true negative rate (*t*_*n*_). True positive rate in this case is defined as the fraction of correctly identified healthy cases out of the actual healthy cases. Similarly, true negative rate is defined as the fraction of the correctly identified unhealthy cases out of the actual unhealthy cases. We then define the accuracy of the algorithm to be: Accuracy = *t*_*p*_ × *t*_*n*_. This definition of the accuracy makes sure that both cases are predicted reasonably accurately since even if one of them is low the accuracy becomes low. In particular, if the algorithm labels all cases to be of the single group, the accuracy becomes zero. The value of the accuracy also depends on how the data is split and so, we average the value over ten random realizations of the splitting. These average accuracies, calculated for *α*_1_-*γ*_1_ planes, as a function of size of the training set for different channels are shown in Fig. 10. It can be seen that even for low amount of training data, the accuracy is quite high.

### Analysis with beat replicated data

The results described in the previous section show that the multifractal analysis is extremely successful for separating healthy and unhealthy classes. Thus, given an ECG time series of a person that is not known to be in one or the other group a priori, we can calculate the corresponding *α*_1_-*γ*_1_ values and then using its location in this parameter plane, we can predict the class the person belongs to with sufficient confidence.

We now present another approach where we invoke the concept of complexity variability^49^. This is a finer level of analysis where the multifractal measures of a given ECG are compared with that of beat replicated data generated from a single beat of the same ECG. This would in a way compare each data within itself and the range of variations can be used effectively as a quantifier for normal and unhealthy states of the heart.

**Figure 11:**
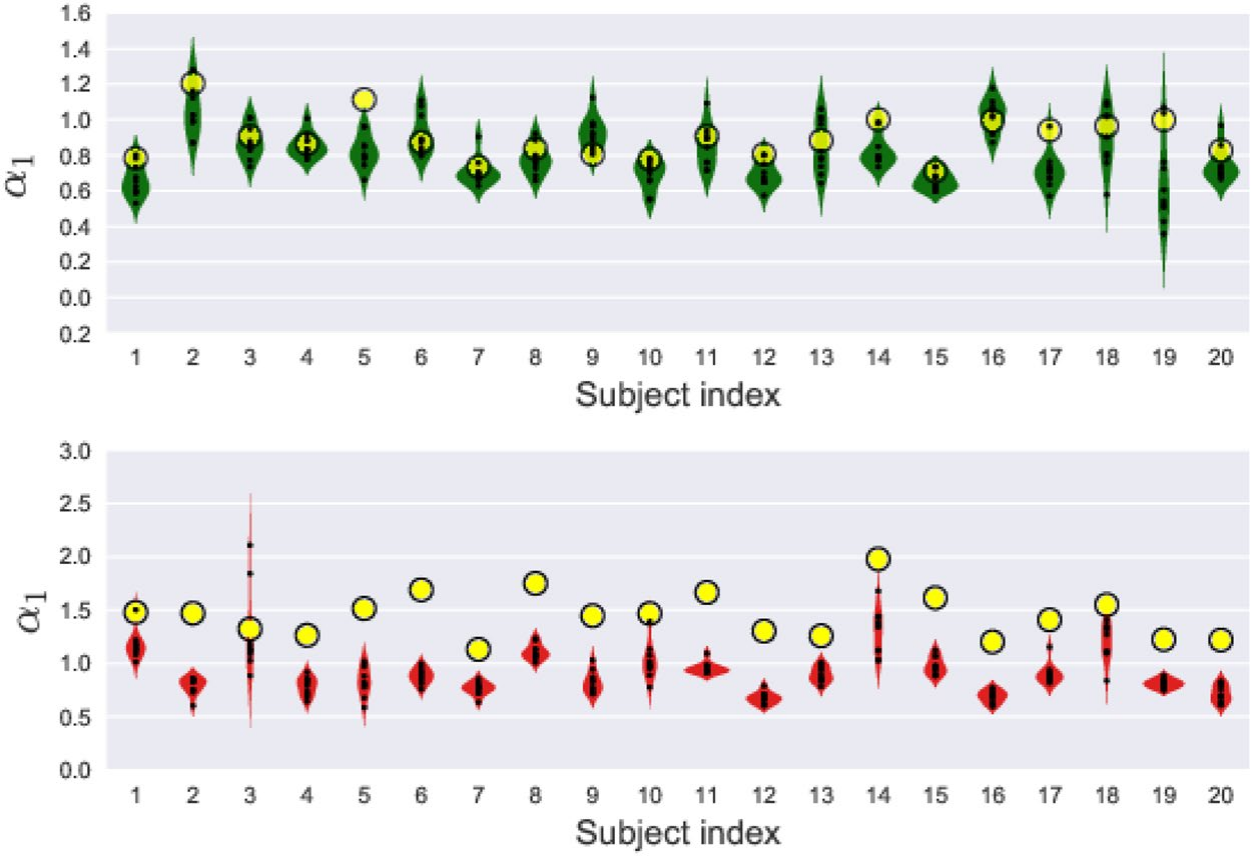
Violin-plot of *α*_1_ values calculated by replicating several randomly chosen beats for few healthy (top panel) and unhealthy (bottom panel) subjects. Each label on *x*-axis represents a subject and the *α*_1_ values for time series obtained by replicating different beats of that person are plotted along *y*-axis. The bigger yellow markers in the plots represent the *α*_1_ values obtained from the complete time series. It can be seen that the actual values for patients tend to fall outside the replicated values.

**Figure 12:**
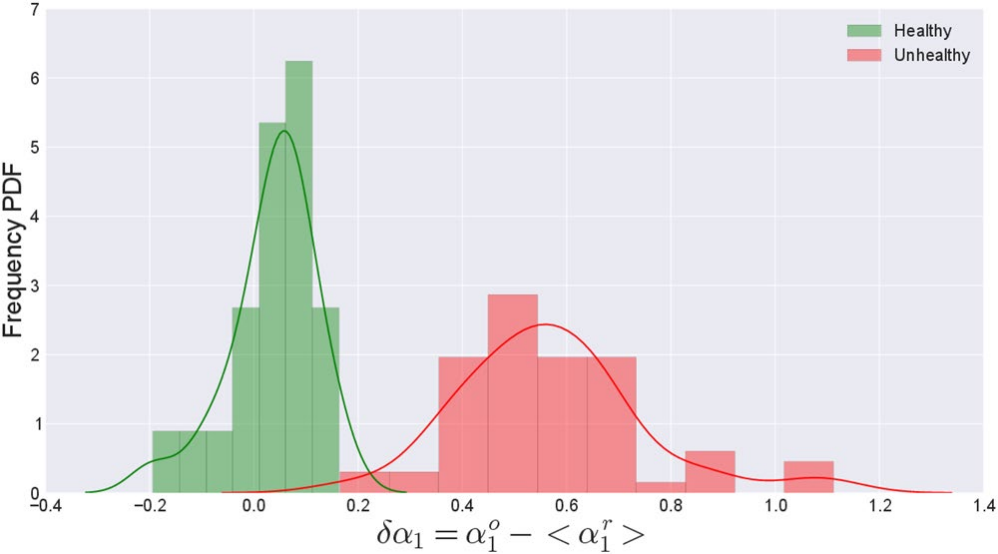
Distributions of the difference between the mean of the replicated values and the *α*_1_ value for the full time series for healthy and unhealthy groups.

For this, we extract 10 different randomly chosen beats from each signal (See Supplementary). Then we replicate each of these beats to get time series of approximately the same sizes as that of the original time series and perform multifractal analysis for each one of these to obtain the parameters *α* and *γ*. For the sake of brevity, we present the results for one of the indices *α*_1_. In Fig. 11, we show the distributions of *α*_1_ values for 20 randomly chosen subjects from each group. For comparison, we also show the original *α*_1_ value in each case (yellow circles). As can be seen, the actual *α*_1_ values in case of healthy subjects tend to coincide with the *α*_1_ values for beat replicated time series. On the other hand, the actual *α*_1_ values in case of unhealthy cases tend to be quite far from the mean of the replicated *α*_1_ values. To make this quantitative, we define *δα*_1_ to be the difference between the *α*_1_ value for the full time series and the mean of the replicated *α*_1_ values as $\delta {\alpha }_{1}={\alpha }_{1}^{o}-\langle {\alpha }_{1}^{r}\rangle$. The histograms of this quantity for the two groups are shown in Fig. 12. It can be seen that the width of the distribution is more for the unhealthy group indicating that the increased variability in the measure can be a useful index in detecting the abnormality.

## Discussion

We report the results of a detailed multifractal analysis of discretized ECG data for two sets of healthy and unhealthy subjects. Our study establishes the highly complex fractal nature of a healthy heart, which gets reduced due to any abnormality in its functions. We could show that the measures derived from the multifractal spectrum can detect abnormalities in heart dynamics with a reasonable level of accuracy. The fact that this is achieved with short time ECG recordings of a significantly small amount of time enhances the scope for its applicability. Moreover the analysis is totally objective with supervised machine learning approach and the results are statistically significant.

The complexity of the ECG signal is understood as due to the adaptability of cardiac activity in response to internal or external stimuli, sympathetic modulations and self-regulations. This greater complexity for normal subjects corresponding to a healthy state for the heart is reflected in the larger values of (*α*_2_ − *α*_1_) in the multifractal spectrum. The presence of unhealthy conditions introduces differences due to defective functions, absence of influence of the autonomic nervous system or regulatory mechanisms that restrict adjustability or variability of heart dynamics. This results in less complexity, requiring only less number of scales to describe the distribution of points in its dynamical attractor in the reconstructed phase space. The reduction in complexity is thus a clear indicator of unhealthy condition and this is being quantified through the analysis reported here. Earlier work using HRV or DFA also capture the complexity in data using (*α*_2_ − *α*_1_) but our analysis use two additional quantifiers, *γ*_1_ and *γ*_2_ representing the shape of the multifractal curve. Since *f*(*α*) represents the scaling of the number of regions with the same *α* on the embedded attractor, these additional indices also have direct implications on the underlying dynamics and its complexity. The variations in this complexity, as carefully characterized in this work, can give clear indications of cardiac malfunctions. The novelty of our approach is that the subtle variations in the shapes of the beats are captured into readable quantities or values which will be much more reliable than conclusions derived from visual inspections. This information can be used to assess the status of health and risk level of the heart easily.

Our analysis, using beat replicated data, helps in comparing quantifiers of one ECG with variations within itself. We find the variance of the measures between actual ECG and the mean value of the same measure obtained from beat replicated data, is more for unhealthy cases. This increased variability can be another clinically meaningful index for detecting abnormalities. In this respect our results are similar to a recently reported study where the complexity variability is considered in the Lyapunov spectra, which is another measure of nonlinearity^49^.

The study is thus an advancement both in the basic understanding of the fractal nature underlying a complex system like heart and its unhealthy states as well as in designing clinically useful practical tools in effective diagnostics and therapy. Understanding and modeling of the intrinsic control mechanisms, nature of nonlinear feedback mechanisms etc will require further detailed studies. The approach of the theory of complex dynamical systems, following the present one, can certainly lead to a more complete description of the mechanisms underlying cardiac activity.

### Data availability statement

All the datasets used in this study are available at www.physionet.org/physiobank/database/ptbdb.

## Electronic supplementary material

Supplementary Information
