# Supplementary material for: Detecting abnormality in heart dynamics from multifractal analysis of ECG signals
Source: Sci Rep. 2017 Nov 9;7:15127. doi: 10.1038/s41598-017-15498-z (PMC5680386; doi:10.1038/s41598-017-15498-z)
Supplement: Supplementary file 1 — Supplementary Information [file 41598_2017_15498_MOESM1_ESM.pdf]

Supplementary information for:

Detecting abnormality in heart dynamics from multifractal analysis of ECG signals

Snehal M. Shekatkar, Yamini Kotriwar, K.P. Harikrishnan, G. Ambika

1 Data acquisition and data description

For our analysis presented here, we have used Data from "PhysioNet" Resource with its PhysioBank archive (https://www.physionet.org/physiobank/database/ptbdb/) [1]. In this link, among the datasets available, we have chosen 129 datasets having the minimum required length. Based on the information available in the corresponding header files, we grouped them into 32 healthy and 92 unhealthy sets. Unhealthy sets are from patients having Myocardial Infraction, Cardiomyopathy, Myocarditis, Dysrhythmia and Hypertrophy. The subjects in the healthy class have age values distributed between 24 and 69 whereas the age values in the unhealthy class are distributed between 41 and 86. Each dataset consists of 15 channels which correspond to different electrodes, the conventional 12 leads $(i, ii, iii, avr, avl, avf, v_1, v_2, v_3, v_4, v_5, v_6)$ together with the 3 Frank leads (v_x, v_y, v_z). Out of these, in this work we concentrate on data from six of the channels v_1 to v_6 that correspond to electrodes which are placed directly on the chest. We expect that the chest electrodes can capture subtle variations in the dynamical response of the heart effectively. Each data corresponds to a real time of 60 seconds and is digitized at 1000 samples per second to obtain in total 60000 points per data.

Among the 97 patient data available to us, 79 suffer from Myocardial Infraction (MI), 6 suffer from Cardiomyopathy, 4 suffer from Myocarditis, 2 suffer from Dysrhythmia and 1 from Hypertrophy while for the remaining 5, the disease information is not available. The subjects in the healthy class have age values distributed between 24 and 69 whereas the age values in the unhealthy class are distributed between 41 and 86.

2 Detrending of the signals

The ECG signals often contain global trends as shown for a typical data in the top panel of Fig. S1. These trends usually are result of the body movement by the subject while the ECG is being taken. As part of the pre processing, we first remove these trends as described below. The de-trended data thus obtained after removing the global trends is shown in the bottom panel of the Fig. S1.

To remove the undesirable trends, we fit a polynomial of a certain degree to the signal, which is then subtracted from the actual signal to get the de-trended signal. To choose the appropriate value of the degree n to be used for the fitting polynomial, we define a deviation $\delta_{(n)}$ of the original signal from the detrended signal as:

$$\delta_{(n)} = \frac{1}{N}\sum_{i=1}^{N}(x_o(i) - x_{(n)}(i))^2 \tag{1}$$

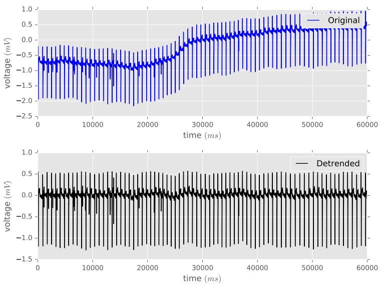

Figure S1: Time series of a randomly chosen subject before detrending (top panel) and after detrending (bottom panel). As explained in the text, the global trend is removed by fitting a polynomial to the original data.

It is important here to choose the right degree for the polynomial to be fit to remove the trend. If the degree of the polynomial is small, it won't be able to remove the higher order trends. Thus, we try different order polynomials to see how they affect the resulting time series. Figure S2 shows the variation of the deviation δ as a function of n, the degree of the polynomial for a few randomly selected ECG time series. As is clear from the plot, values of the deviations saturate for sufficiently high n. Based on this, for all the datasets we use $n = 20$ to detrend them.

3 Embedding and phase space reconstruction

An important way in which our method differs from the other methods for the analysis of ECG waveforms is the use of the embedding technique. Two typical ECG are

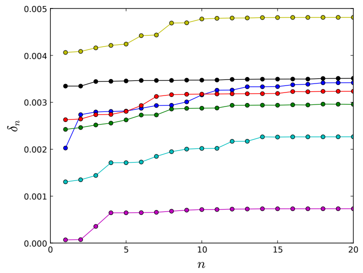

Figure S2: Deviations as a function of the degree of the detrending polynomial for a few randomly selected ECG waveforms. It can be seen that for high values of the degree, deviations saturate.

shown in Figure S3. It is almost impossible to quantify the difference between the variations in these waveforms visually. To resolve this problem, we turn towards a dynamical systems' framework and use these waveforms to reconstruct the dynamical attractors of these systems. By comparing the properties of these reconstructed attractors, we can quantify the differences between the original waveforms. The details of the embedding technique are given below.

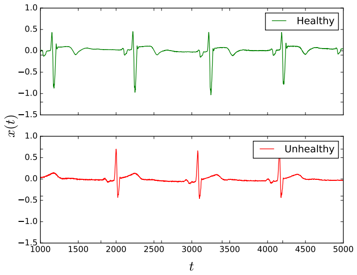

Figure S3: A part of the ECG time series of a typical healthy and unhealthy subjects.

For uniformity, all the values in the time series $c(t_k)$ are first scaled between 0 and 1 by using a transformation of "compression":

$$s(t_k) = \frac{c(t_k) - c_{\text{min}}}{c_{\text{max}} - c_{\text{min}}} \quad (2)$$

where c_{min} and c_{max} are minimum and maximum values in the time series $c(t_k)$ respectively. Each time series $s(t_k)$ is then embedded into an M dimensional space, by constructing vectors as:

$$\vec{x}_i = [s(t_i), s(t_i+\tau), s(t_i+2\tau), \cdots, s(t_i+(M-1)\tau)] \quad (3)$$

Here a time delay τ is the time, measured in units of sampling rate $\triangle = t_{i+1} - t_i$, at which autocorrelation of the signal falls to $1/e$ of its original value [2]. It is easy to see that there are in total $N - (M-1)\tau$ embedded vectors. As mentioned in the main text, the value of M is chosen from the saturation values of the correlation dimension. Taken's embedding theorem dictates that the phase space trajectories or attractor obtained from these vectors have the same topological properties as that of the original system[3].

The distribution of the points on the attractor thus reconstructed, is usually non-uniform and determines the multifractal properties of the attractor. Figure S4 shows the multifractal spectra for the time series given in Figure S3. Our method uses four parameters α_1, α_2, γ_1 and γ_2 to characterize a given multifractal spectrum uniquely. As explained in the main text, the difference $\alpha_2 - \alpha_1$ or the width of the multifractal spectrum, is a measure of the complexity of the attractor since it represents the range of the scales required to characterize the attractor fully. The other two indices γ_1 and γ_2 represent the functional form (in the Eq.(3) from the main text) of the multifractal curves and are required for the complete characterization. It can thus be seen from Figure S4 that the waveform for the healthy heart is more complex than that of the unhealthy heart.

To see this in detail for many datasets together, one can visualize the various parameter planes to see if the healthy and unhealthy groups cluster in the different regions of these planes. As an example, we show the results for α_1-α_2 planes in Figure S5 and almost all healthy cases are seen to be more complex than the unhealthy cases.

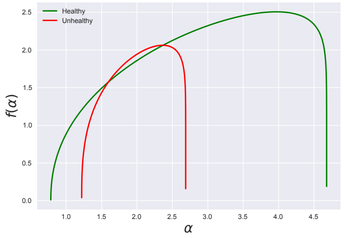

Figure S4: Multifractal spectra for the two time series shown in Figure S3. The spectrum for the healthy case can be seen to be broader than the unhealthy case.

As explained in the main text, the α_2 values have larger numerical errors for the datasets used in the study. Hence for further characterization, we rely on α_1-γ_1 and α_1-α_0 planes and show how they can be effectively used to group the datasets as healthy and unhealthy.

Extracting a single beat from an ECG time series

Identifying a single beat in an ECG signal is tricky since a beat cannot be defined as a pattern that repeats with

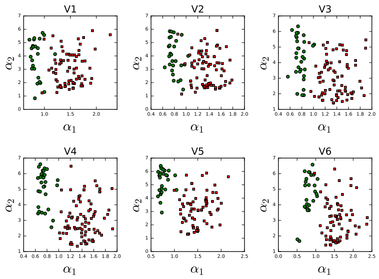

Figure S5: α_1-α_2 planes for different channels. The red squares represent the patients and green circles represent the healthy subjects.

exact periodicity in the ECG signal. However, it is easy to see that ECG signals do have a certain approximate periodicity because of the presence of beats. For the data used, in units of milliseconds, the individual beats are seen to repeat with a period $T \in (600, 1500)$. To find the exact value of the period, we calculate the autocorrelation of the time series as a function of the lag τ. The resulting plot for a typical ECG time series is shown in Figure S6. The τ value corresponding to the highest peak in this range is then taken to be the period of the signal and the same is used to extract a single beat from the time series.

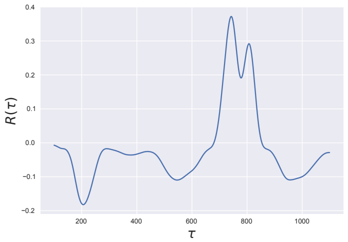

Figure S6: Autocorrelation as a function of the time lag τ for a typical ECG waveform. The range of the time lag values is chosen so that the approximate period falls in this range. The τ at which autocorrelation becomes highest is then taken as the period of the ECG waveform.

The beats thus extracted are used for generating the beat replicated data used in the analysis.

References

[1] Goldberger, A. L. *et al.* Physiobank, physiotoolkit, and physionet. *Circulation* **101**, 215 (2000).

[2] Harikrishnan, K. P., Misra, R., Ambika, G. & Kembhavi, A. K. A non-subjective approach to the gp algorithm for analysing noisy time series. *Physica D: Nonlinear Phenomena* **215**, 137 (2006).

[3] Takens, F. *et al.* Dynamical systems and turbulence. *Lecture notes in mathematics* **898**, 366 (1981).
